# Muscular Dystrophy and Heart Failure: An Unusual Association

**DOI:** 10.7759/cureus.18604

**Published:** 2021-10-08

**Authors:** João P Pais, Marta B Sousa, Ana R Cambão, Ana Nascimento, Diana Guerra

**Affiliations:** 1 Internal Medicine, Unidade Local de Saúde do Alto Minho (ULSAM) - Hospital de Santa Lúzia, Viana do Castelo, PRT

**Keywords:** myotonic dystrophy, cardiovascular disease, neuromuscular disease, genetic disease, heart failure

## Abstract

Type one muscular dystrophy (DM1) is the most common inherited muscular dystrophy in the adult population. Typically, DM1 presents as myotonia, muscle weakness, cataracts, and cardiac abnormalities, mainly in the conduction system. Although left ventricular dysfunction is not the most common manifestation of DM1, it can be seen with disease progression. The presentation of DM1 as a de novo heart failure is unusual, making its diagnosis a clinical challenge.

## Introduction

Type one muscular dystrophy (DM1) is the most common inherited muscular dystrophy in the adult population, with an estimated prevalence of 1/8000 [1]. DM1 is caused by the expansion of a repetitive nucleotide segment in the 3’ untranslated region of the myotonic dystrophy protein kinase (DMPK). The clinical presentation of DM1 is divided into four main categories, based on the age of onset and symptoms presented: congenital, childhood, classical (adult onset), and mild (late onset) [2]. Typically, DM1 presents as myotonia, muscle weakness, cataracts, and cardiac abnormalities with the latter being the main cause of mortality in this population. The most prevalent cardiac manifestations are related to the conduction system and range from benign first-degree atrioventricular (AV) block to malignant ventricular arrhythmias [3]. Although heart failure symptoms are not the most common manifestation of DM1, it can be seen with disease progression and has a prevalence estimated from 7.2% to 11.3% [4]. Some individuals and families can present with clinical findings related to heart failure, with an echocardiogram revealing a dilated cardiomyopathy [1]. 

## Case presentation

A 71-year-old male patient presented to the emergency department (ED) with asthenia, loss of appetite, and nonquantified weight loss evolving, at least, over the last six months. Associated with these constitutional symptoms, the patient also mentioned dyspnea for progressively smaller efforts, which impacted the patient’s autonomy and quality of life. At the observation, he was only able to walk from the bed to a chair, with grade III functional capacity according to the New York Heart Association (NYHA). More recently, he presented episodes of paroxysmal nocturnal dyspnea associated with orthopnea that appeared for the first time three months prior to observation, with complaints of occasional dysphagia for solid and liquid food. No other neurological symptoms were present at this time. At this point, the patient consulted a cardiologist, and a transthoracic echocardiogram revealed a left ventricular dysfunction with a left ventricular ejection fraction (LVEF) of 36%. Coronary angiography was also performed, which showed no significant coronary artery lesions. One week prior to observation in ED, the patient started complaining of dyspnea at rest with a dry cough. A diagnosis of respiratory infection was assumed, and antibiotic treatment with levofloxacin was started, with no clinically relevant improvements, which lead the patient to observation in our ED. During disease progression, there was no history of fever, syncope, chest pain, or palpitations.

Regarding past medical history, he was a previous smoker, had permanent atrial fibrillation, was hypocoagulated with apixaban, and had stage IIIA chronic kidney disease according to the "Kidney Disease: Improving Global Outcomes" (KDIGO) classification.

On physical examination, the patient had a peripheral oxygen saturation of 92%, with normal values for blood pressure and heart rate. Jugular venous pressure was present at 45º. Cardiac auscultation was arrhythmic, and pulmonary auscultation revealed inspiratory crackles in the lower half of both lungs. There was no peripheral edema.

**Figure 1:**
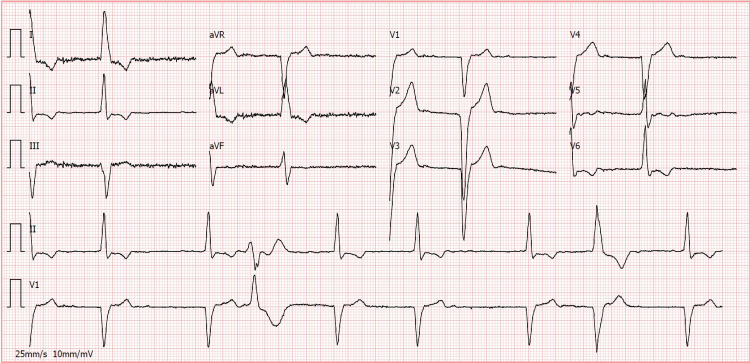
The 12-lead electrocardiogram (EKG) obtained in the emergency department showing atrial fibrillation and a complete left bundle branch block

A 12-lead electrocardiogram (EKG) was obtained, which showed atrial fibrillation, with a heart rate of 49 bpm, and a complete left bundle branch block (Figure 1).

An arterial blood gas test revealed a hypoxemic respiratory failure, with a partial oxygen pressure of 55 mmHg. The remaining blood tests showed a hypernatremia of 149 mmol/L and an elevated B-type natriuretic peptide (BNP) of 754.9 pg/mL. The remaining results of the blood tests can be seen in Table 1.

**Table 1:** Blood tests performed in the emergency department

Hemoglobin	13.0 g/dL
Leukocyte	1.05 x 10^9^/L
Platelet	162 x 10^9^/L
Urea	65 mg/dL
Creatinine	1.36 mg/dL
Na^+^	149 mmol/L
K^+^	4.4 mmol/L
Total bilirubin	1.08 mg/dL
Direct bilirubin	0.49 mg/dL
Lactic dehydrogenase	193 IU/L
Alkaline phosphatase	103 IU/L
Gamma-glutamyl transferase	55 IU/L
Alanine transaminase	27 IU/L
C-reactive protein	0.92 mg/dL
Myoglobin	254 ng/mL
High-sensitivity troponin I	36 pg/mL
B-type natriuretic peptide	754.9 pg/mL
INR	1.75

**Figure 2:**
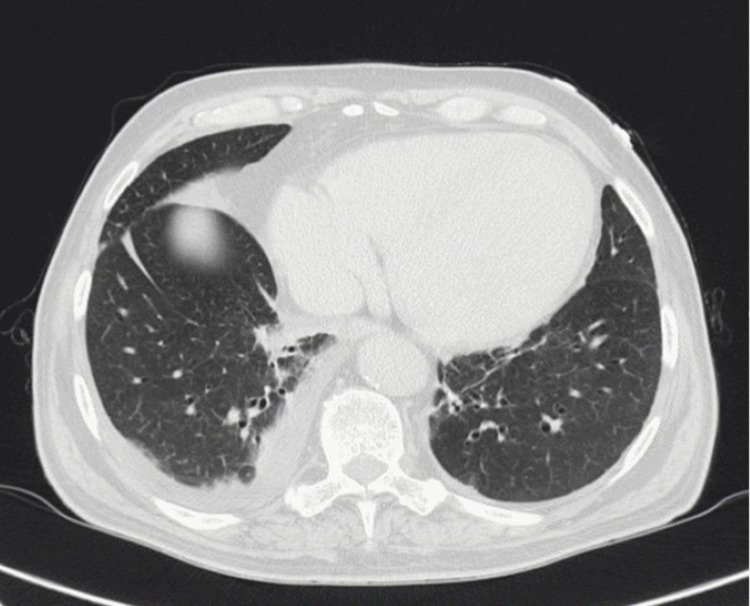
Thoracic CT scan revealing cardiomegaly and pleural effusion on the right lung

**Figure 3:**
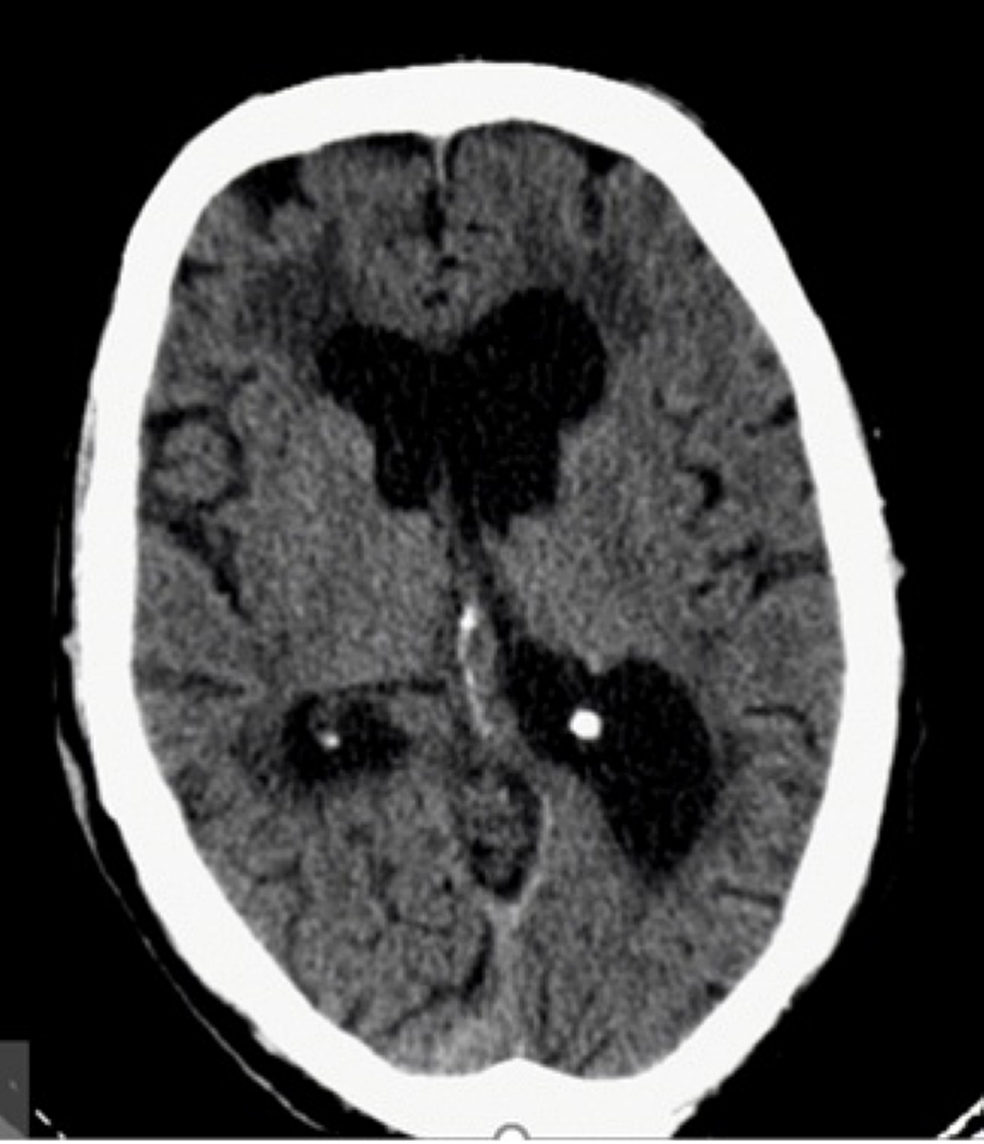
Cranioencephalic CT revealing a cerebral atrophy with subcortical predominance

A thoracic computerized tomography (CT) scan was performed and showed a significant cardiomegaly, right pleural effusion, and ground-glass opacities in both lungs (Figure 2). A cranioencephalic CT scan was also performed, revealing a cerebral atrophy with a subcortical pattern (Figure 3).

The patient was admitted for study and symptomatic treatment, assuming the diagnosis of acute heart failure with reduced ejection fraction (HFrEF) of unknown etiology, associated with constitutional symptoms and occasional dysphagia.

**Figure 4:**
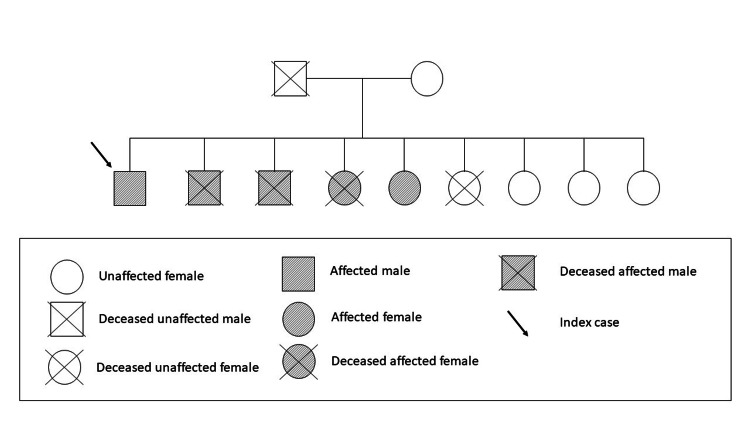
Family tree showing index case and family members affected by DM1

We initiated treatment with intravenous furosemide and started prognostic modifying drugs for HFrEF, with progressive improvement of the congestive symptoms. However, he developed a hypercapnic respiratory insufficiency. Respiratory function tests were made, revealing a restrictive ventilatory pattern. A cervical CT scan was also made, revealing no significant alterations. At this point, the suspicion of a neuromuscular disease began to rise, and an electromyography test was performed, showing signs of active denervation and reinnervation in three body segments (cranial, cervical, and lumbar). With a more thorough clinical interview, a family history of DM1 was identified (Figure 4), and genetic testing for this disease revealed a pathogenic expansion of the DMPK gene, with a trinucleotide cytosine-thymine-guanine (CTG) repeat size of 312. A diagnostic of DM1 was made in this patient. After stabilization, the patient was discharged to the outpatient setting for follow-up. In the outpatient setting, a cardiac magnetic resonance was made, which identified a dilated cardiomyopathy and alterations suggestive of previous ischemic events in the medial third of the inferior wall of the left ventricle and in the septal wall. Cardiac catheterization was repeated, maintaining no significant coronary lesions. Regarding the findings in the complementary examinations, a diagnostic of a dilated cardiomyopathy in the context of DM1 was made.

## Discussion

Although being the most common muscular dystrophy in the adult population, the presentation described in this clinical case is infrequent. Classically, patients present with complaints of muscle weakness, mainly in the distal musculature, associated with myotonia [5]. In this clinical case, the main complaints presented by the patient (dyspnea, paroxysmal nocturnal dyspnea, and orthopnea) were more suggestive of a diagnosis of heart failure. The absence of complaints of muscle weakness and myotonia is not typical at the presentation of DM1, which made the diagnosis more difficult to attain. Additionally, the cardiac involvement in DM1 typically is associated with abnormalities in the conduction system, ranging from first-degree atrioventricular blocks to episodes of sudden death [2]. Only a small fraction of patients with DM1 present with left ventricular dysfunction, being usually subclinical [4]. The presence of clinically relevant ventricular dysfunction is uncommon in patients with DM1 mainly because this dysfunction is more prevalent in more advanced phases of the disease when the daily activity of the patients is greatly impaired and oxygen demand is diminished [6]. In our patient, the signs and symptoms of heart failure were predominant, with the presence of dysphagia being the only hint of the presence of a neuromuscular disease. The positive familial history was only described by the patient when the suspicion of a neuromuscular disease was already present. The presentation of DM1 as a decompensated de novo heart failure in a patient with minor neuromuscular involvement is a rare occurrence. In our clinical case, the cardiac magnetic resonance showed alterations suggestive of ischemic disease; however, cardiac catheterization showed no coronary lesions. These findings further fundament the diagnostic of a myotonic heart disease. Typically, these patients do not present coronary disease, and cardiac magnetic resonance usually shows fibrotic lesions, mainly in the interventricular septum and mid-myocardium [6], which are difficult to differentiate from the lesions observed after an ischemic event.

## Conclusions

Being a disease that mainly affects the neuromuscular system, DM1 can also have a significant impact on other organs, such as the heart. The most common alterations in the cardiovascular system refer to the conduction system, varying from atrioventricular blocks to episodes of sudden death. Although the presence of symptomatic heart failure is a rare event in the natural history of patients with DM1, it is important to recognize it as a group of symptoms that directly relate to disease progression and a worse prognosis for these patients.
